# Supplementary material for: Cyclo-stationary distributions of mRNA and Protein counts for random cell division times
Source: bioRxiv. 2025 Jun 8:2025.06.06.658238. Preprint. [Version 1] doi: 10.1101/2025.06.06.658238 (PMC12157499; doi:10.1101/2025.06.06.658238)
Supplement: Supplement 1 [file media-1.pdf]

Supplementary Material

Cyclo-stationary distributions of mRNA and Protein counts for random cell division times

Syed Yunus Ali,[1, *] Aditya Saran,[1] Ashok Prasad,[2, †] Abhyudai Singh,[3, ‡] and Dibyendu Das[1, 4, §]

[1] *Department of Physics, Indian Institute of Technology Bombay, Powai, Mumbai 400076, India*
[2] *School of Biomedical and Chemical Engineering, Colorado State University, Fort Collins, Colorado 80521, USA*
[3] *Department of Electrical and Computer Engineering, University of Delaware, Newark, DE 19716, USA*
[4] *Max Planck Institute for the Physics of Complex Systems, 01187 Dresden, Germany*

* syed_24@iitb.ac.in
† ashok.prasad@colostate.edu
‡ absingh@udel.edu
§ dibyendudas@iitb.ac.in

I. GENERAL FRAMEWORK TO STUDY THE CYCLO-STATIONARY DISTRIBUTIONS

A. Equation for the cyclo-stationary copy number distributions $P_+^{ss}(y_+)$ and $P_-^{ss}(y_-)$

In the main text, we present the equations for $P_+^i(y_{+,i}, t_{s_i})$ and $P_-^i(y_{-,i}, t_{s_i})$, the distributions of copy numbers just after and just before the ith cell division respectively, as follows:

$$P_+^i(y_{+,i}, t_{s_i}) = \sum_{y_{+,i-1}} \sum_{x_{+,i}} B(y_{+,i} + x_{+,i}, \frac{1}{2}, x_{+,i}) p(y_{+,i} + x_{+,i}, t_{s_i} | y_{+,i-1})\ P_+^{i-1}(y_{+,i-1}, t_{s_{i-1}}), \tag{1}$$

$$P_-^i(y_{-,i}, t_{s_i}) = \sum_{y_{+,i-1}} p(y_{-,i}, t_{s_i} | y_{+,i-1})\ P_+^{i-1}(y_{+,i-1}, t_{s_{i-1}}). \tag{2}$$

Integrating Eq. 1 over the joint probability distribution of $g_2(t_{s_i}, t_{s_{i-1}})$ of successive division time intervals,

$$\int_0^\infty \int_0^\infty dt_{s_i} dt_{s_{i-1}} g_2(t_{s_i}, t_{s_{i-1}}) P_+^i(y_{+,i}, t_{s_i}) = \int_0^\infty \int_0^\infty dt_{s_i} dt_{s_{i-1}} g_2(t_{s_i}, t_{s_{i-1}}) \sum_{y_{+,i-1}} \sum_{x_{+,i}} B(y_{+,i} + x_{+,i}, \frac{1}{2}, x_{+,i})$$
$$p(y_{+,i} + x_{+,i} | y_{+,i-1}, t_{s_i}) P_+^i(y_{+,i-1}, t_{s_{i-1}}). \tag{3}$$

If it is further assumed that successive division times are uncorrelated, i.e. $g_2(t_{s_i}, t_{s_{i-1}}) = g(t_{s_i}) g(t_{s_{i-1}})$, where $g(t_{s_i})$ is the normalized distributions of t_{s_i}, we have

$$\int_0^\infty \int_0^\infty dt_{s_i} dt_{s_{i-1}} g(t_{s_i}) g(t_{s_{i-1}}) P_+^i(y_{+,i}, t_{s_i}) = \int_0^\infty \int_0^\infty dt_{s_i} dt_{s_{i-1}} g(t_{s_i}) g(t_{s_{i-1}}) \sum_{y_{+,i-1}} \sum_{x_{+,i}} B(y_{+,i} + x_{+,i}, \frac{1}{2}, x_{+,i})$$
$$p(y_{+,i} + x_{+,i} | y_{+,i-1}, t_{s_i}) P_+^i(y_{+,i-1}, t_{s_{i-1}}) \tag{4}$$

which simplifies to

$$\int_0^\infty dt_{s_i} g(t_{s_i}) P_+^i(y_{+,i}, t_{s_i}) = \sum_{y_{+,i-1}, x_{+,i}} \int_0^\infty dt_{s_i} g(t_{s_i}) p(y_{+,i} + x_{+,i} | y_{+,i-1}, t_{s_i})\ B\left(y_{+,i} + x_{+,i}, \frac{1}{2}, x_{+,i}\right)$$
$$\times \int_0^\infty dt_{s_{i-1}} g(t_{s_{i-1}}) P_+^i(y_{+,i-1}, t_{s_{i-1}}) \tag{5}$$

For $i \gg 1$, as the cyclo-stationary regime is attained, we may define the distribution as cell birth, $P_+^{ss}(y_+) = \int_0^\infty dt_{s_i} g(t_{s_i}) P_+^i(y_{+,i}, t_{s_i})$. Dropping the subscripts i, and setting $t_{s_i} = t_s$ and $y_{+,i-1} = y'_+$, Eq. 5 gives

$$P_+^{ss}(y_+) = \sum_{y'_+} \sum_{x_+} \int_0^\infty dt_s g(t_s)\ B(y_+ + x_+, \frac{1}{2}, x_+) \times p(y_+ + x_+, t_s | y'_+)\ P_+^{ss}(y'_+). \tag{6}$$

In a similar way, one may derive from Eq. 2 above, the cyclo-stationary distribution just before division, defined as $P_-^{ss}(y_-) = \int_0^\infty dt_{s_i} g(t_{s_i}) P_-^i(y_{-,i}, t_{s_i})$, related to $P_+^{ss}(y_+)$:

$$P_-^{ss}(y_-) = \sum_{y'_+} \int_0^\infty dt_s g(t_s)\ p(y_-, t_s | y'_+, t_s)\ P_+^{ss}(y'_+). \tag{7}$$

Eqs. 6 and 7 are the two Eqs. 3 and 4 in the main text.

B. Self-consistent integral for the generating functions $F_+(q)$ and its relation to $F_-(q)$

We define generating function $F_\pm(q) = \sum_{y_\pm=0}^\infty q^{y_\pm} P_\pm^{ss}(y_\pm)$. Multiply $\sum_{y_+} q^{y_+}$ on both sides of Eq. 6 we get,

$$F_+(q) = \sum_{y'_+} \sum_{x_+} \int_0^\infty dt_s g(t_s) \sum_{y_+} q^{y_+} p(y_+ + x_+, t_s | y'_+)\ B(y_+ + x_+, \frac{1}{2}, x_+) P_+^{ss}(y'_+) \tag{8}$$

We defining a new variable $\tilde{y}_+ = y_+ + x_+$, where $x_+ \le \tilde{y}_+ \le \infty$. But since $B(\tilde{y}_+, \frac{1}{2}, x_+) = 0$ for $\tilde{y}_+ < x_+$, we put $\tilde{y}_+ \in [0, \infty)$. Using the explicit form of the binomial function, the recursion relation of $F_+(q)$ may then be written as:

$$\begin{aligned}
F_+(q) &= \sum_{y'_+} \int_0^\infty dt_s g(t_s) \sum_{\tilde{y}_+} q^{\tilde{y}_+} p(\tilde{y}_+, t_s|y'_+) \sum_{x_+=0}^{\tilde{y}_+} \frac{1}{q^{x_+}} \binom{\tilde{y}_+}{x_+} \left(\frac{1}{2}\right)^{\tilde{y}_+ - x_+} \left(\frac{1}{2}\right)^{x_+} P_+^{ss}(y'_+) \\
&= \sum_{y'_+} \int_0^\infty dt_s g(t_s) \left(\sum_{\tilde{y}_+} q^{\tilde{y}_+} p(\tilde{y}_+, t_s|y'_+) \left(\frac{q+1}{2q}\right)^{\tilde{y}_+} \right) P_+^{ss}(y'_+) \\
&= \int_0^\infty dt_s g(t_s) \sum_{y'_+} F\left(\frac{q+1}{2}, t_s \middle| y'_+\right) P_+^{ss}(y'_+). \qquad (9)
\end{aligned}$$

Here $F(q,t|y') = \sum_y q^y p(y,t|y')$ is the generating function of the probability $p(y,t|y')$ tied to the process of gene expression. Let us assume that this generating function has a form $F(q,t|y') = \mathcal{H}(q-1, \gamma_y t) \times (1 + (q-1)e^{-\gamma_y t})^{y'}$. This leads to the following self-consistent integral for $F_+(q)$ (which is Eq. 6 in the main text):

$$F_+(q) = \int_0^\infty dt_s g(t_s) \mathcal{H}(\frac{q-1}{2}, \gamma_y t_s) F_+\left(1 + \frac{(q-1)}{2} e^{-\gamma_y t_s}\right). \qquad (10)$$

In a similar way, by multiplying $\sum_{y_-} q^{y_-}$ on both sides of Eq. 7 we get,

$$\begin{aligned}
F_-(q) &= \sum_{y_-} q^{y_-} \sum_{y'_+} \int_0^\infty dt_s g(t_s) \times p(y_-, t_s|y'_+) \times P_+^{ss}(y'_+) \\
&= \int_0^\infty dt_s g(t_s) \sum_{y'_+} F(q, t_s|y'_+) P_+^{ss}(y'_+) \\
&= \int_0^\infty dt_s g(t_s) \mathcal{H}(q-1, \gamma_y t_s) F_+\left(1 + (q-1)e^{-\gamma_y t_s}\right). \qquad (11)
\end{aligned}$$

Putting $q = 2q' - 1$ in Eq. 10 we have,

$$F_+(2q'-1) = \int_0^\infty dt_s g(t_s) \mathcal{H}(q'-1, \gamma_y t_s) F_+\left(1 + (q'-1)e^{-\gamma_y t_s}\right), \qquad (12)$$

and comparing with Eq. 11, we obtain (the Eq. 7 in the main text):

$$F_-(q) = F_+(2q-1) \qquad (13)$$

C. Closed form of $F_+(q)$ for Fixed cell cycle times, and the intractable nested integrals for Random cell cycle times

For fixed cell division times, i.e. $g(t_s) = \delta(t_s - T)$, Eq. 10 reduces to

$$F_+(q) = \mathcal{H}\left(\frac{q-1}{2}, \gamma_y T\right) F_+\left(1 + \frac{(q-1)}{2} e^{-\gamma_y T}\right). \qquad (14)$$

If we set, $q - 1 = w$ then Eq. 14 becomes to

$$F_+(1+w) = \mathcal{H}\left(\frac{w}{2}, \gamma_y T\right) F_+\left(1 + \frac{w}{2} e^{-\gamma_y T}\right). \qquad (15)$$

This recursion formula may be iterated to obtain

$$F_+(1+w) = \prod_{k=1}^{j} \mathcal{H}\left(\frac{w}{2} \frac{e^{-(k-1)\gamma_y T}}{2^{k-1}}, \gamma_y T\right) F_+\left(1 + w\left(\frac{e^{-\gamma_y T}}{2}\right)^j\right). \qquad (16)$$

As $j \to \infty$, $F_+\left(1 + w\left(\frac{e^{-\gamma_y T}}{2}\right)^j\right) \to F_+(1) = 1$, and hence

$$F_+(1+w) = \prod_{k=1}^{\infty} \mathcal{H}\left(\frac{w}{2}\frac{e^{-(k-1)\gamma_y T}}{2^{k-1}}, \gamma_y T\right). \tag{17}$$

Replacing back $w = q - 1$ Eq. 17 gives the closed form in Eq. 8 in the main text.

For random division times t_s, with any general normalised function $g(t_s)$, when we substitute $w = q - 1$, Eq. 10 becomes:

$$F_+(1+w) = \int_0^{\infty} dt_s\ g(t_s)\mathcal{H}\left(\frac{w}{2}, \gamma_y t_s\right) F_+\left(1 + \frac{w}{2}e^{-\gamma_y t_s}\right). \tag{18}$$

Iterating one step, and replacing the F_+ on the right side with an similar integral as Eq. 18, we obtain:

$$F_+(w+1) = \int_0^{\infty} dt_s^1\ g(t_s^1)\mathcal{H}\left(\frac{w}{2}, \gamma_y t_s^1\right) \int_0^{\infty} dt_s^2\ g(t_s^2)\mathcal{H}\left(\frac{w_1(t_s^1)}{2}, \gamma_y t_s^2\right) F_+\left(1 + \frac{w_1(t_s^1)}{2}e^{-\gamma_y t_s^2}\right), \tag{19}$$

where $w_1(t_s^1) = \frac{w}{2}e^{-\gamma_y t_s^1}$. Continuing with the next iteration,

$$F_+(w+1) = \int_0^{\infty} dt_s^1\ g(t_s^1)\mathcal{H}\left(\frac{w}{2}, \gamma_y t_s^1\right) \int_0^{\infty} dt_s^2\ g(t_s^2)\mathcal{H}\left(\frac{w_1}{2}, \gamma_y t_s^2\right) \int_0^{\infty} dt_s^3\ g(t_s^3)\mathcal{H}\left(\frac{w_2}{2}, \gamma_y t_s^3\right) F_+\left(1 + \frac{w_2}{2}e^{-\gamma_y t_s^3}\right), \tag{20}$$

where $w_2 = w_2(t_s^1, t_s^2) = \frac{w_1}{2}e^{-\gamma_y t_s^2} = \frac{w}{2^2}e^{-\gamma_y t_s^1}e^{-\gamma_y t_s^2}$. Repeating this indefinitely, as $j \to \infty$ we have $F_+(1 + w_j) \to F_+(1) = 1$, where $w_j = \frac{w_{j-1}}{2}e^{-\gamma_y t_s^j}$, and hence

$$F_+(1+w) = \prod_{k=1}^{\infty} \int_0^{\infty} dt_s^k\ g(t_s^k)\mathcal{H}\left(\frac{w_{k-1}}{2}, \gamma_y t_s^k\right). \tag{21}$$

As $w_k = w_k(t_s^1, t_s^2, ..., t_s^k) = \frac{w}{2^k}e^{-\gamma_y t_s^1}e^{-\gamma_y t_s^2}...e^{-\gamma_y t_s^k}$, the above nested integrals are in general intractable. This is why the problem has stayed challenging.

D. Deriving the cyclo-stationary distributions at birth and before division, from the series expansion of generating function about $q = 1$

Although $P_+^{ss}(y_+)$ are themselves coefficients of the series expansion of $F_+(q)$ about $q = 0$, we may start with an alternate expansion of $F_+(q) = \sum_{k=0}^{\infty} \frac{(q-1)^k}{k!} F_+^{(k)}(1)$ about $q = 1$. In that case,

$$P_+^{ss}(y_+) = \frac{1}{y_+!}\left[\frac{\partial^{y_+}}{\partial q^{y_+}} F_+(q)\right]_{q=0} = \frac{1}{y_+!}\left[\frac{\partial^{y_+}}{\partial q^{y_+}} \sum_{k=0}^{\infty} \frac{(q-1)^k}{k!} F_+^{(k)}(1)\right]_{q=0} = \sum_{k=y_+}^{\infty} \binom{k}{y_+} \frac{(-1)^{k-y_+}}{k!} F_+^{(k)}(1) \tag{22}$$

Similarly, using Eq. 13, we have

$$\begin{aligned} P_-^{ss}(y_-) = \frac{1}{y_-!}\left[\frac{\partial^{y_-}}{\partial q^{y_-}} F_-(q)\right]_{q=0} &= \frac{1}{y_-!}\left[\frac{\partial^{y_-}}{\partial q^{y_-}} F_+(2q-1)\right]_{q=0} = \frac{1}{y_-!}\left[\frac{\partial^{y_-}}{\partial q^{y_-}} \sum_{k=0}^{\infty} \frac{2^k(q-1)^k}{k!} F_+^{(k)}(1)\bigg|_{q=0}\right] \\ &= \sum_{k=y_-}^{\infty} \binom{k}{y_-} \frac{(-1)^{k-y_-}}{k!} 2^k F_+^{(k)}(1) \end{aligned} \tag{23}$$

Thus above, we have the series expansions of $P_+^{ss}(y_+)$ and $P_-^{ss}(y_-)$ (Eq. 11 and 12 in the main text) involving the coefficients $F_+^{(k)}(1)$.

E. The first three cumulants of $P_+^{ss}(y_+)$ in terms of the coefficients $F_+^{(k)}(1)$

Using $F_+(q) = \sum_{y_+} P_+^{ss}(y_+) q^{y_+} = \sum_{k=0}^{\infty} \frac{(q-1)^k}{k!} F_+^{(k)}(1)$, we may obtain the cumulants as follows. The mean of y_+:

$$\langle y_+ \rangle = \sum_{y_+} y_+ P_+^{ss}(y_+) = q\frac{\partial}{\partial q} F_+(q)\Big|_{q=1} = q\frac{\partial}{\partial q}\left(\sum_k \frac{(q-1)^k}{k!} F_+^{(k)}(1)\right)_{q=1} = q\sum_k \frac{k(q-1)^{k-1}}{k!} F_+^{(k)}(1)\Big|_{q=1} = F_+^{(1)}(1) \tag{24}$$

The second moment

$$\langle y_+^2 \rangle = q\frac{\partial}{\partial q} q\frac{\partial}{\partial q} F_+(q)\Big|_{q=1} = q\sum_k \frac{k(q-1)^{k-1}}{k!} F_+^{(k)}(1) + q^2 \sum_k \frac{k(k-1)(q-1)^{k-2}}{k!} F_+^{(k)}(1)\Big|_{q=1} = F_+^{(1)}(1) + F_+^{(2)}(1) \tag{25}$$

Hence the Variance

$$\kappa_2 = \langle y_+^2 \rangle - \langle y_+ \rangle^2 = F_+^{(1)}(1) + F_+^{(2)}(1) - \left(F_+^{(1)}(1)\right)^2 \tag{26}$$

The third moment

$$\begin{aligned}\langle y_+^3 \rangle &= q\frac{d}{dq} q\frac{d}{dq} q\frac{d}{dq} F_+(q)\Big|_{q=1} \\ &= q\sum_k \frac{k(q-1)^{k-1}}{k!} F_+^{(k)}(1) + 3q^2 \sum_k \frac{k(k-1)(q-1)^{k-2}}{k!} F_+^{(k)}(1) + q^2 \sum_k \frac{k(k-1)(k-2)(q-1)^{k-3}}{k!} F_+^{(k)}(1)\Big|_{q=1} \\ &= F_+^{(1)}(1) + 3F_+^{(2)}(1) + F_+^{(3)}(1)\end{aligned} \tag{27}$$

Hence the third cumulant is (see Eqs. 24, 26 and 27 above)

$$\kappa_3 = \langle (y_+ - \kappa_1)^3 \rangle = [F_+^{(1)}(1) + 3F_+^{(2)}(1) + F_+^{(3)}(1)] - 3\kappa_1\kappa_2 - \kappa_1^3 \tag{28}$$

The above equations appear as Eq. 13, 14 and 15 in the main text. Using the above cumulants we obtain $CV^2 = \kappa_2/\kappa_1^2$ and Skewness $= \kappa_3/\kappa_2^{3/2}$ in our study.

II. STATISTICS OF THE mRNA NUMBER IN THE CYCLO-STATIONARY STATE

A. The generating function related to the model of transcription, and thereby obtaining function $\mathcal{H}$

The Master equation for the stochastic model of mRNA production and degradation is

$$\frac{dp(m,t|m'_+)}{dt} = k_m p(m-1,t|m'_+) + \gamma_m(m+1)p(m+1,t|m'_+) - (\gamma_m m + k_m)p(m,t|m'_+). \tag{29}$$

Here k_m is the transcription rate, and γ_m is the degradation rate of mRNAs. The generating function $F(q,t) = \sum_{j=0}^{\infty} q^m p(m,t|m'_+)$ of the distribution $p(m,t|m'_+)$ satisfies (using Eq. 29 above) the following:

$$\frac{\partial F(q,t)}{\partial t} + \gamma_m(q-1)\frac{\partial F}{\partial q} = k_m(q-1)F. \tag{30}$$

Eq. 30 can be solved by using the method of Lagrange characteristic, and one gets

$$F(q,t) = e^{\lambda(t)(q-1)}(1 + (q-1)e^{-\gamma_m t})^{m'_+}, \tag{31}$$

where $\lambda(t) = (k_m/\gamma_m)[1 - e^{-\gamma_m t}]$. For brevity we will use $\lambda(t) \equiv \lambda$ below. Thus comparing with Eq. 5 of the main text (also see below Eq. 9), we identify the function

$$\mathcal{H} = e^{\frac{k_m}{\gamma_m}[1-e^{-\gamma_m t}](q-1)}. \tag{32}$$

B. Obtaining the coefficients $F_+^{(k)}(1)$ and the series of the distributions $P_\pm^{ss}(m_\pm)$

Using $\mathcal{H}$ from Eq. 32 in Eq. 10, and $F_+(q) = \sum_{j=0}^{\infty} \frac{(q-1)^j}{j!} F_+^{(j)}(1)$ we have

$$\begin{aligned} F_+(q) &= \int_0^\infty dt_s g(t_s) e^{\lambda((q-1)/2)} F_+(1 + ((q-1)/2)e^{-\gamma_m t_s}) \\ &= \int_0^\infty dt_s g(t_s) e^{\lambda((q-1)/2)} \sum_{j=0}^{\infty} \frac{F_+^{(j)}(1)}{j!} \left(\frac{q-1}{2}\right)^j e^{-j\gamma_m t_s} \\ &= \int_0^\infty dt_s g(t_s) \sum_{l=0}^{\infty}\sum_{j=0}^{\infty} \frac{\lambda^l \left(\frac{q-1}{2}\right)^l}{l!} \frac{F_+^{(j)}(1)}{j!} \left(\frac{q-1}{2}\right)^j e^{-j\gamma_m t_s} \\ &= \int_0^\infty dt_s g(t_s) \sum_{l=0}^{\infty}\sum_{j=0}^{\infty} \frac{F_+^{(j)}(1)}{l!j!} \left(\frac{q-1}{2}\right)^{l+j} \left(\frac{k_m}{\gamma_m}\right)^l e^{-j\gamma_m t_s}(1 - e^{-\gamma_m t_s})^l \end{aligned} \tag{33}$$

Changing summation indices to $k = l + j$ and defining $\Psi_{k,j} = \int_0^\infty dt_s g(t_s) e^{-j\gamma_m t_s}(1 - e^{-\gamma_m t_s})^{k-j}$, Eq. 33 becomes

$$F_+(q) = \sum_{k=0}^{\infty} \frac{1}{k!} \left(\frac{q-1}{2}\right)^k \sum_{j=0}^{k} \binom{k}{j} \left(\frac{k_m}{\gamma_m}\right)^{k-j} \Psi_{k,j} F_+^{(j)}(1) \tag{34}$$

Using the relation $F_+(q) = \sum_{k=0}^{\infty} \frac{(q-1)^k}{k!} F_+^k(1)$ on the left side of Eq. 34 above, and comparing coefficients we get the desired recursion relation (which appears in Eq. 21 of the main text):

$$F_+^k(1) = \frac{1}{2^k} \sum_{j=0}^{k} \left(\frac{k_m}{\gamma_m}\right)^{k-j} \binom{k}{j} \Psi_{k,j} F_+^j(1). \tag{35}$$

The first few coefficients are explicitly as follows. As $\sum_{m_+} P^{ss}(m_+) = 1$ we firstly have $F_+^{(0)}(1) = 1$. The next coefficient (from Eq. 35) is

$$F_+^1(1) = \frac{1}{2}\left(\Psi_{1,0}\frac{k_m}{\gamma_m} + \Psi_{1,1} F_+^{(1)}(1)\right) = \frac{\frac{k_m}{\gamma_m}\frac{1}{2}\Psi_{1,0}}{1 - \frac{1}{2}\Psi_{1,1}} \tag{36}$$

Proceeding similarly we have $F_+^{(2)}(1)$ determined by $F_+^{(1)}(1)$ as follows:

$$F_+^{(2)}(1) = \frac{\left(\frac{k_m}{\gamma_m}\right)^2 \frac{1}{2^2}\Psi_{2,0}}{1 - \frac{1}{2^2}\Psi_{2,2}} + \frac{\left(\frac{k_m}{\gamma_m}\right)^2 \frac{1}{2^3}\binom{2}{1}\Psi_{2,1}\Psi_{1,0}}{(1 - \frac{1}{2}\Psi_{1,1})(1 - \frac{1}{2^2}\Psi_{2,2})} \tag{37}$$

Next, the coefficient

$$\begin{aligned} F^3(1) = &\left[\frac{\frac{1}{2^3}\left(\frac{k_m}{\gamma_m}\right)^3 \Psi_{3,0}}{1 - \frac{1}{2^3}\Psi_{3,3}}\right] + \left[\frac{\frac{1}{2^4}\binom{3}{1}\Psi_{3,1}\Psi_{1,0}\left(\frac{k_m}{\gamma_m}\right)^3}{(1 - \frac{1}{2^3}\Psi_{3,3})(1 - \frac{1}{2}\Psi_{1,1})}\right] + \left[\frac{\frac{1}{2^5}\binom{3}{2}\Psi_{3,2}\Psi_{2,0}\left(\frac{k_m}{\gamma_m}\right)^3}{(1 - \frac{1}{2^2}\Psi_{2,2})(1 - \frac{1}{2^3}\Psi_{3,3})}\right] \\ &+ \left[\frac{\frac{1}{2^6}\binom{3}{2}\Psi_{3,2}\Psi_{2,1}\Psi_{1,0}\left(\frac{k_m}{\gamma_m}\right)^3}{(1 - \frac{1}{2}\Psi_{1,1})(1 - (\frac{1}{2^2}\Psi_{2,2})(1 - \frac{1}{2^3}\Psi_{3,3})}\right] \end{aligned} \tag{38}$$

Observing the pattern of the successive coefficients, we obtain the general solution for $F_+^{(k)}(1)$ as follows:

$$F_+^{(k)}(1) = \left(\frac{k_m}{\gamma_m}\right)^k \frac{1}{2^k} \frac{1}{(1 - \frac{1}{2^k}\Psi_{k,k})} \left[\sum_{\{S_{k-1}\}} \frac{(\frac{1}{2})^{\sum_i j_i} \phi_{k,j_z}\ \phi_{j_z,j_{z-1}} \ldots \phi_{j_{1,0}}}{\prod_i (1 - \frac{1}{2^{j_i}}\Psi_{j_i,j_i})} + \phi_{k,0}\right] \tag{39}$$

where $\phi_{k,j} = \Psi_{k,j}\binom{k}{j}$. Here $\{S_{k-1}\}$ denotes the set of all the subsets $S_{k-1} = \{j_i\} = (j_z, j_{z-1}...j_1)$ of integers $j_i \in (1, 2, ...k-1)$ such that $j_z > j_{z-1} > ... > j_1$. For example for $k = 3$, the subsets are (1), (2), and (2,1) as is seen in Eq. 38.

With the coefficients given by Eq. 39, the cyclo-stationary distributions are formally given by the series:

$$P_+^{ss}(m_+) = \sum_{k=m_+}^{\infty} \binom{k}{m_+} \frac{(-1)^{k-m_+}}{k!} F_+^{(k)}(1) \tag{40}$$

$$P_-^{ss}(m_-) = \sum_{k=m_-}^{\infty} \binom{k}{m_-} \frac{(-1)^{k-m_-} 2^k}{k!} F_+^{(k)}(1) \tag{41}$$

C. The cyclo-stationary mRNA distributions are Poisson for fixed cell-division times T

For fixed cell division times, $g(t_s) = \delta(t_s - T)$, we have $\Psi_{k,j} = e^{-j\gamma_m T}(1 - e^{-\gamma_m T})^{k-j}$, and

$$\begin{aligned}\Psi_{k,j_z}\Psi_{j_z,j_{z-1}}\dots\Psi_{j_1,0} &= e^{-j_z\gamma_m T}(1-e^{-\gamma_m T})^{k-j_z} e^{-j_{z-1}\gamma_m T}(1-e^{-\gamma_m T})^{j_z - j_{z-1}} .... e^{-0*\gamma_m T}(1-e^{-\gamma_m T})^{j_1} \\ &= e^{-\gamma_m T \sum_{i=1}^{z} j_i}(1-e^{-\gamma_m T})^k \end{aligned} \tag{42}$$

Consequently from Eqs. 40 and 39,

$$\begin{aligned} P_+^{ss}(m_+) &= \sum_{k=m_+}^{\infty} \frac{(-1)^{k-m_+}}{k!}\binom{k}{m_+} F_+^{(k)}(1) \\ &= \sum_{k=m_+}^{\infty} \frac{(-1)^{j-m_+}}{k!}\binom{k}{m_+}\left[\left(\frac{k_m}{\gamma_m}\right)^k \frac{1}{2^k}\frac{1}{(1-\frac{1}{2^k}\Psi_{k,k})}\left[\sum_{\{S_{k-1}\}} \frac{(\frac{1}{2})^{\sum j_i}\phi_{k,j_z}\phi_{j_z,j_{z-1}}...\phi_{j_1,0}}{\prod_i\left(1-\frac{1}{2^{j_i}}\Psi_{j_i,j_i}\right)} + \phi_{k,0}\right]\right] \\ &= \sum_{k=m_+}^{\infty} \frac{(-1)^{k-m_+}}{j!}\binom{k}{m_+}\left(\frac{k_m}{\gamma_m}\right)^k \frac{\frac{1}{2^k}}{1-\frac{1}{2^k}\Psi_{k,k}}(1-e^{-\gamma_m T})^k \left[1 + \sum_{\{S_{k-1}\}}\binom{k}{j_z}\binom{j_z}{j_{z-1}}...\binom{j_2}{j_1}\frac{\prod_i\left(\frac{1}{2}e^{-\gamma_m T}\right)^{j_i}}{\prod_i\left(1-(\frac{1}{2}e^{-\gamma_m T})^{j_i}\right)}\right] \end{aligned} \tag{43}$$

Using the the following identity [1], with $x = \frac{1}{2}e^{-\gamma_m T}$ in this case,

$$1 + \sum_{\{S_{k-1}\}}\binom{k}{j_z}\binom{j_z}{j_{z-1}}...\binom{j_2}{j_1}\prod_i \frac{x^{j_i}}{1-x^{j_i}} = \frac{1-x^k}{(1-x)^k} \tag{44}$$

Thus the $(1 - x^k)$ factors cancel from the numerator and denominator, and Eq. 43 simplifies to

$$\begin{aligned} P_{ss}(m_+) &= \sum_{k=m_+}^{\infty}\binom{k}{m_+}\frac{(-1)^{k-m_+}}{k!}\left(\frac{k_m}{\gamma_m}\right)^k \frac{\frac{1}{2^k}}{(1-e^{-\gamma_m T}\frac{1}{2})^k}(1-e^{-\gamma_m T})^k \\ &= \sum_{k=m_+}^{\infty}\frac{d^k}{m_+!(k-m_+)!}(-1)^{k-m_+} \end{aligned} \tag{45}$$

with $d = \left(\frac{k_m}{\gamma_m}\right)\frac{(1-e^{-\gamma_m T})}{(2-e^{-\gamma_m T})}$. Thus $F_+^{(k)}(1) = d^k$. Replacing $k - m_+ = r$, then Eq. 45 reduces to a Poisson distribution:

$$P_+^{ss}(m_+) = \frac{d^{m_+}}{m_+!}\sum_{r=0}^{\infty}\frac{(-1)^r d^r}{r!} = \frac{d^{m_+}}{m_+!}\exp(-d). \tag{46}$$

Since Eq. 41 has an extra factor of 2^k multiplying $F_+^{(k)}(1)$, we would have d replaced by $2d$ and the distribution:

$$P_-^{ss}(m_-) = \frac{(2d)^{m_-}}{m_-!}\exp(-2d). \tag{47}$$

D. Simplified form of the cyclo-stationary distributions for Exponentially distributed cell cycle times

For exponentially distributed division times, $g(t_s) = \lambda e^{-\lambda t_s}$ (with $\lambda = 1/T$), and

$$\Psi_{k,j} = \int_0^\infty dt_s g(t_s) e^{-\gamma_m t_s}(1-e^{-\gamma_m t_s})^{k-j} = \lambda/\gamma_m\, B\left(\frac{\lambda + j\gamma_m}{\gamma_m}, k-j+1\right) = \frac{\lambda}{\gamma_m}\frac{\Gamma(k-j+1)\Gamma\left(\frac{\lambda}{\gamma_m}+j\right)}{\Gamma\left(\frac{\lambda}{\gamma_m}+k+1\right)} \tag{48}$$

and $\Psi_{k,k} = \frac{\lambda}{\gamma_m}\frac{\Gamma(1)\Gamma(\frac{\lambda}{\gamma_m}+k)}{\Gamma(\frac{\lambda}{\gamma_m}+k+1)} = \frac{\lambda}{\lambda+\gamma_m k}$. Then

$$\phi_{k,j} = \binom{k}{j}\Psi_{k,j} = \left(\frac{\lambda}{\gamma_m}\right)\frac{\Gamma(k+1)\Gamma[\frac{\lambda}{\gamma_m}+j]}{\Gamma(j+1)\Gamma[\frac{\lambda}{\gamma_m}+k+1]} \tag{49}$$

Hence $\phi_{k,0} = k!\frac{\Gamma(\frac{\lambda}{\gamma_m})}{\Gamma(\frac{\lambda}{\gamma_m}+k+1)}$. Furthermore,

$$\phi_{n,j_z}\ \phi_{j_z,j_{z-1}}\ \ldots\ \phi_{j_1,0} = \frac{\lambda^{z+1}}{\gamma_m^{z+1}}\frac{k!\ \Gamma[\frac{\lambda}{\gamma_m}]}{\Gamma[\frac{\lambda}{\gamma_m}+k+1]}\prod_{i=1}\frac{\gamma_m}{\lambda+\gamma_m j_i} \tag{50}$$

Substituting the above, in Eq. 40 and 39, we have

$$\begin{aligned}
P_+^{ss}(m_+) &= \sum_{k=m_+}^\infty \binom{k}{m_+}(-1)^{k-m_+}\frac{1}{k!}\left(\frac{k_m}{\gamma_m}\right)^k \frac{1/2^k}{(1-1/2^k\Psi_{k,k})}\left[\sum_{\{S_{k-1}\}}\frac{(\frac{1}{2})^{\sum j_i}\phi_{k,j_z}\ \phi_{j_z,j_{z-1}}\ \ldots\ \phi_{j_1,0}}{\prod_i(1-\frac{1}{2^{j_i}}\Psi_{j_i,j_i})}+\phi_{k,0}\right]\\
&= \sum_{k=m_+}^\infty \binom{k}{m_+}(-1)^{k-m_+}\frac{\frac{1}{2^k}\left(\frac{k_m}{\gamma_m}\right)^k}{(1-\frac{1}{2^k}\Psi_{k,k})}\left[\frac{\lambda}{\gamma_m}\frac{\Gamma(\frac{\lambda}{\gamma_m})}{\Gamma(\frac{\lambda}{\gamma_m}+k+1)}+\sum_{\{S_{k-1}\}}\frac{(\frac{1}{2})^{\sum j_i}\frac{\lambda^{z+1}}{\gamma_m^{z+1}}\frac{\Gamma(\lambda/\gamma_m)}{\Gamma(\lambda/\gamma_m+k+1)}}{\prod_i(1-\frac{1}{2^{j_i}}\Psi_{j_i,j_i})}\prod_i\frac{\gamma_m}{\lambda+\gamma_m j_i}\right]\\
&= \sum_{k=m_+}^\infty \binom{k}{m_+}(-1)^{k-m_+}\frac{\frac{1}{2^k}\left(\frac{k_m}{\gamma_m}\right)^k}{(1-\frac{1}{2^k}\Psi_{k,k})}\frac{\Gamma(\frac{\lambda}{\gamma_m}+1)}{\Gamma(\frac{\lambda}{\gamma_m}+k+1)}\left[1+\sum_{\{S_{k-1}\}}\prod_i\frac{\frac{1}{2^{j_i}}\Psi_{j_i,j_i}}{(1-\frac{1}{2^{j_i}}\Psi_{j_i,j_i})}\right]\\
&= \sum_{k=m_+}^\infty \binom{k}{m_+}(-1)^{k-m_+}\frac{\frac{1}{2^k}\left(\frac{k_m}{\gamma_m}\right)^k}{(1-\frac{1}{2^k}\Psi_{k,k})}\frac{\Gamma(\frac{\lambda}{\gamma_m}+1)}{\Gamma(\frac{\lambda}{\gamma_m}+k+1)}\frac{1}{\prod_i^{k-1}(1-\frac{1}{2^i}\Psi_{i,i})}\\
&= \sum_{k=m_+}^\infty \binom{k}{m_+}\frac{(-1)^{k-m_+}}{k!}\frac{k!\left(\frac{k_m}{2}\right)^k}{\prod_i^k(\lambda+\gamma_m i-\frac{1}{2^i}\lambda)},
\end{aligned} \tag{51}$$

where we have used an identity [1], with $f(j_i) = \frac{1}{2^{j_i}}\Psi_{j_i,j_i}$ as follows:

$$1+\sum_{\{S_{k-1}\}}\prod_i\frac{f(j_i)}{(1-f(j_i))} = \frac{1}{\prod_i^{k-1}(1-f(i))} \tag{52}$$

Eq. 51 shows that $F_+^{(k)}(1) = k!\left(\frac{k_m}{2}\right)^k/\prod_i^k(\lambda+\gamma_m i-\frac{1}{2^i}\lambda)$. For obtaining the distribution before division, we note the extra factor of 2^k in Eq. 41, and that implies (comparing with Eq. 51)

$$P_-^{ss}(m_-) = \sum_{k=m_-}^\infty \binom{k}{m_-}\frac{(-1)^{k-m_-}}{k!}\frac{k!\,(k_m)^k}{\prod_i^k(\lambda+\gamma_m i-\frac{1}{2^i}\lambda)}. \tag{53}$$

E. Expressions of CV^2 and Skewness of the distribution $P_+^{ss}(m_+)$ of mRNA at cell birth.

Using the exact expressions of $F_+^{(1)}(1)$, $F_+^{(2)}(1)$, and $F_+^{(3)}(1)$ in Eqs. 36, 37 and 38, we have obtained the cumulants (from Eqs. 24, 26 and 28), and thus studied the mean, CV^2 and Skewness in the main text.

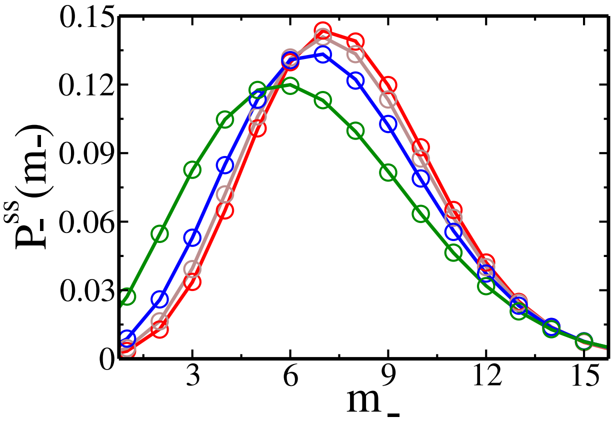

FIG. 1. Cyclo-stationary distribution $P_{-}^{ss}(m_{-})$ of mRNA for the four $g(t_s)$ shown in Fig. 3 in the main text (corresponding colours being the same)

III. STATISTICS OF CYCLO-STATIONARY PROTEIN COUNT

A. The generating function for protein kinetics, and thereby obtaining function $\mathcal{H}$

The Master equation for the bursty protein translation is the following (with initial copy number being n'_{+}):

$$\frac{\partial P(n,t|n'_{+})}{\partial t} = k_m \left[\sum_{r=1}^{n} \frac{b^r}{(b+1)^{r+1}} P(n-r,t|n'_{+}) - \frac{b}{b+1} P(n,t|n'_{+})\right] + \gamma_p \left[(n+1)P(n+1,t|n'_{+}) - nP(n,t|n'_{+})\right]. \tag{54}$$

The generating function $F(q,t|n'_{+}) = \sum_{j=0}^{\infty} q^n p(n,t|n'_{+})$ is known to satisfy [2]

$$\frac{1}{v}\frac{\partial F}{\partial \tau} + \frac{\partial F}{\partial v} = \frac{ab}{1-bv} F \tag{55}$$

where $v = q-1$, $\tau = \gamma_p t$ and $a = \frac{k_m}{\gamma_p}$. The solution of Eq. 54 by the method of Lagrange characteristics yield [2]

$$F(q,t|n'_{+}) = \left(\frac{1-b(q-1)e^{-\gamma_p t_s}}{1-b(q-1)}\right)^a \times \left(1+(q-1)\,e^{-\gamma_p t_s}\right)^{n'_{+}}. \tag{56}$$

Thus comparing with Eq. 5 of the main text (also see below Eq. 9), we identify the function

$$\mathcal{H} = \left(\frac{1-b(q-1)e^{-\gamma_p t_s}}{1-b(q-1)}\right)^a. \tag{57}$$

B. Obtaining the recursion relation for the coefficients $F_+^{(k)}(1)$ in the series of the distributions $P_\pm^{ss}(m_\pm)$

Using $\mathcal{H}$ from Eq. 57 in Eq. 10, and $F_+(q) = \sum_{j=0}^{\infty} \frac{(q-1)^j}{j!} F_+^{(j)}(1)$ we have

$$\begin{aligned}
F_+(q) &= \int_0^\infty dt_s g(t_s) \left(\frac{1 - \frac{b}{2}(q-1) e^{-\gamma_p t_s}}{1 - \frac{b}{2}(q-1)} \right)^a F_+ \left(1 + \frac{(q-1)}{2} e^{-\gamma_p t_s} \right) \\
&= \int_0^\infty dt_s g(t_s) \left(\frac{1 - \frac{b}{2}(q-1) e^{-\gamma_p t_s}}{1 - \frac{b}{2}(q-1)} \right)^a \sum_{j=0}^{\infty} \frac{F_+^j(1)\ (q-1)^j}{2^j j!} e^{-j\gamma_p t_s} \\
&= \int_0^\infty dt_s g(t_s) \sum_{l=0}^{\infty} \frac{\Gamma(a+1)}{\Gamma(a-l+1)} \left(\frac{b}{2}\right)^l \frac{(-1)^l (q-1)^l e^{-l\gamma_p t_s}}{l!} \sum_{s=0}^{\infty} \frac{\Gamma(a+s)}{\Gamma(a)} \left(\frac{b}{2}\right)^s \frac{(q-1)^s}{s!} \sum_{j=0}^{\infty} \frac{F_+^j(1) e^{-j\gamma_p t_s} (q-1)^j}{2^j j!} \\
&= \sum_{l=0}^{\infty}\sum_{j=0}^{\infty}\sum_{s=0}^{\infty} \int_0^\infty dt_s g(t_s) (-1)^l \frac{\Gamma(a+1)}{\Gamma(a-l+1)} \frac{\Gamma(a+s)}{\Gamma(a)} \left(\frac{b}{2}\right)^{l+s} (q-1)^{l+j+s} e^{-(l+j)\gamma_p t_s} \binom{a+k-1}{k} \frac{F_+^{(j)}(1)}{2^j\ l!\ s!\ j!}
\end{aligned} \tag{58}$$

We define $L_{l+j} = \int_0^\infty dt_s g(t_s) e^{-(l+j)\gamma_p t_s}$, and replace indices $l + j + s = k$, which leads to

$$F_+(q) = \sum_{k=0}^{\infty} \frac{(q-1)^k}{k!} \sum_{l=0}^{k}\sum_{j=0}^{k-l} a \frac{k!\Gamma(a+k-l-j)}{\Gamma(a-l+1)} (-1)^l \left(\frac{b}{2}\right)^{k-j} \frac{L_{l+j} F_+^{(j)}(1)}{l! j! (k-l-j)! 2^j} \tag{59}$$

A series expansion on the left side of Eq. 59 about $q = 1$, and comparing with the right side, yields the desired recursion relation:

$$F_+^{(k)}(1) = ak! \sum_{l=0}^{k}\sum_{j=0}^{k-l} (-1)^l \frac{b^{k-j}}{2^k} L_{l+j} \frac{(a+k-l-j-1)!}{(a-l)!\ l!\ j!\ (k-l-j)!} F_+^{(j)}(1) \tag{60}$$

The above Eq. 60 appears as Eq. 26 in the main text. Once these coefficients $F_+^{(k)}(1)$ are solved for, they may be used to obtain the cyclo-stationary distributions

$$P_+^{(ss)}(n_+) = \sum_{k=n_+}^{\infty} \binom{k}{n_+} \frac{(-1)^{k-n_+}}{k!} F_+^{(k)}(1) \tag{61}$$

$$P_-^{(ss)}(n_-) = \sum_{k=n_-}^{\infty} \binom{k}{n_-} \frac{(-1)^{k-n_-} 2^k}{k!} F_+^{(k)}(1) \tag{62}$$

C. Expressions of first three $F_+^{(k)}(1)$ which determine exactly CV^2 and Skewness of the distribution $P_+^{ss}(n_+)$ of protein.

Firstly, $F_+^{(0)}(1) = 1$. Then using the above Eq. 60 recursively, we get

$$F_+^{(1)}(1) = ab \frac{1 - L_1}{2 - L_1} \tag{63}$$

then,

$$F_+^{(2)}(1) = \frac{ab^2}{4 - L_2} \left[(1 - L_2) + a \frac{(2 - 3L_1 + L_1 L_2)}{(2 - L_1)} \right] \tag{64}$$

and then,

$$\begin{aligned}
F_+^{(3)}(1) = \frac{1}{8 - L_3} \Big[& ab^3 \Big((1+a)(2+a) - 3a((1+a)L_1 - (a-1)L_2) - (a-2)(a-1)L_3 \Big) \\
& + 3ab^2 \Big((1+a)L_1 - 2aL_2 + (a-1)L_3 \Big) F_+^{(1)}(1) + 3ab \Big(L_2 - L_3 \Big) F_+^{(2)}(1) \Big]
\end{aligned} \tag{65}$$

The constants L_1, L_2 and L_3 may be evaluated given a $g(t_s)$. Then the above Eqs. 63, 64 and 65 are substituted in the Eqs. 24, 26 and 28, to obtain the cumulants and thus CV^2 and Skewness, which are studied in the main text.

IV. COMPUTATIONAL METHODS

A. Precautions to perform numerical sums of different series to obtain the coefficients $F_+^{(k)}(1)$ and the theoretical cyclo-stationary distributions $P_\pm^{ss}$

In this work we had to sum various series to obtain the desired coefficients and functions.

The equations for the coefficients $F_+^{(k)}(1)$ appear as Eq. 35 for mRNA, and Eq. 60 for protein, and are of the form

$$F_+^{(k)}(1) = \sum_{j=1}^{k-1} c_{k,j} F_+^{(j)}(1) \tag{66}$$

As the values of $F_+^{(j)}(1)$ grow very fast with j we loose precision soon in ordinary calculations. A better way to store large numbers is by taking logarithm, and we do so for terms in Eq. 66. Thus we store terms

$$u_{k,j} = \ln c_{k,j} + \ln F_+^{(j)}(1). \tag{67}$$

We specify very high precision for such calculation and storage in Mathematica (through the SetPrecision[d] command) up to $d = 100$ decimal places in case of mRNA and $d = 200$ decimal places for protein. We reconstruct back the coefficient

$$F_+^{(k)}(1) = \sum_{j=1}^{k-1} e^{u_{k,j}}. \tag{68}$$

Once the coefficients $F_+^{(k)}(1)$ are obtained by the above method, up to some desired k, we put them in the series in Eqs. 40 and 41 for mRNA, and Eqs. 61, 62 for protein, to obatin the cyclo-stationary distributions. For mRNA, convergence was attained for $\sim 30-50$ terms in the series of $P_\pm^{ss}(m_\pm)$.

For protein, ordinary sum of the series of $P_\pm^{ss}(n_\pm)$ were not enough with reasonable values of k. We used Borel sum formula as follows:

$$P_\pm^{ss}(n_\pm) = \sum_{k=n_\pm}^{\infty} f(k, n_\pm) = P_\pm^{ss}(n_\pm)\Big|_{\text{Borel}} = \lim_{t\to\infty} e^{-t} \sum_{n=0}^{M\to\infty} \frac{t^n}{n!} \sum_{k'=0}^{n} f(k' + n_\pm, n_\pm) \tag{69}$$

In calculations we choose $M \sim 200-250$ and $t = 30$ to obtain convergence of the protein cyclo-stationary distributions.

B. Kinetic Monte Carlo Simulations

We perform Kinetic Monte Carlo (KMC) or Gillespie [3] simulations for the various models governing the transcription or translation models of mRNA and protein this paper, undergoing Binomial partitioning after random time intervals t_s drawn from some distribution $g(t_s)$. Thus, at any instant, there are three possible events to either increase, decrease, or reset the copy number (due to cell partition). We typically use $\sim 10^7$ histories for getting the data for various distributions and cumulants, which were then compared with the theory.

V. AGE DEPENDENT CYCLO-STATIONARY DISTRIBUTIONS

The cyclo-stationary distribution $P^{ss}(y, \tau)$ of cells at an age τ before the next cell division, may written with respect to $P_+^{ss}(y_+)$ at birth, as follows:

$$P^{ss}(y, \tau) = \sum_{y_+} P_+^{ss}(y_+) p(y, \tau | y_+). \tag{70}$$

Using its generating function $\tilde{G}(q,\tau) = \sum_y q^y P^{ss}(y,\tau)$, from Eq. 70 (and using the same steps as in Eqs. 9 and 10)

$$\tilde{G}(q,\tau) = \sum_{y_+} P_+^{ss}(y_+) F(q,\tau|y_+) = \mathcal{H}(q-1,\gamma_y\tau)\ F_+(1+(q-1)e^{-\gamma_y\tau})$$
$$= \sum_k \frac{(q-1)^k}{k!} G_y^{(k)}(\tau) \tag{71}$$

The defined quantities $G_y^{(k)}(\tau)$ are obtained in Eq. 71 by expressing $F_+(q) = \sum_{j=0}^{\infty} \frac{(q-1)^j}{j!} F_+^{(j)}(1)$, and expanding $\mathcal{H}$ as a power series of $(q-1)$. The resulting expression of $G_y^{(k)}(\tau)$ are of the form of the integrands of Eqs. 33 or 58 (without the $\int_0^\infty dt_S g(t_s)/2^k$ factors), and are explicitly given for mRNA and protein in Eqs. 30 and 31 in the main text. Finally it is easy to obtain the desired age-dependent distributions in terms of $G_y^{(k)}(\tau)$ as

$$P^{ss}(y,\tau) = \frac{1}{y!}\frac{\partial^y \tilde{G}}{\partial q^y}\bigg|_{q=0} = \sum_{k=y}^{\infty} \binom{k}{y} \frac{(-1)^{k-y}}{k!} G_y^{(k)}(\tau). \tag{72}$$

VI. GENERATING FUNCTIONS OF PROTEIN DISTRIBUTION AT BIRTH, FOR DETERMINISTIC PARTITIONING, AND DETERMINISTIC GENE EXPRESSION

If we have a deterministic partitioning, we would replace the binomial distribution $B(\tilde{y}_+, \frac{1}{2}, x_+)$ in Eqs. 8 and 9 by $\delta_{x_+,\tilde{y}_+/2}$ and as a result

$$F_+(q) = \sum_{y'_+} \int_0^\infty dt_s g(t_s) \sum_{\tilde{y}_+} q^{\tilde{y}_+} p(\tilde{y}_+, t_s|y'_+) \sum_{x_+=0}^{\tilde{y}_+} \frac{1}{q^{x_+}} \delta_{x_+,\frac{\tilde{y}_+}{2}} P_+^{ss}(y'_+)$$
$$= \sum_{y'_+} \int_0^\infty dt_s g(t_s) \left(\sum_{\tilde{y}_+} q^{\tilde{y}_+/2} p(\tilde{y}_+, t_s|y'_+) \right) P_+^{ss}(y'_+)$$
$$= \int_0^\infty dt_s g(t_s) \sum_{y'_+} F\left(\sqrt{q}, t_s \middle| y'_+\right) P_+^{ss}(y'_+). \tag{73}$$

For proteins $y \equiv n$, and using the appropriate $F(q,t|n'_+)$ from Eq. 56, we have the counterpart of Eq. 10 as:

$$F_+(q) = \int_0^\infty dt_s g(t_s) \left(\frac{1 - b\left(\sqrt{q}-1\right) e^{-\gamma_p t_s}}{1 - b\left(\sqrt{q}-1\right)} \right)^a F_+\left(1 + (\sqrt{q}-1)e^{-\gamma_p t_s}\right) \tag{74}$$

If in addition to deterministic partitioning, one also has deterministic protein kinetics

$$\frac{dn}{dt} = k_m b - \gamma_p n, \tag{75}$$

then $\tilde{n}_+ = n'_+ e^{-\gamma_p t_s} + ab(1 - e^{-\gamma_p t_s})$ and F in Eq. 73 gets replaced by $q^{\frac{1}{2}(\lambda_p + n'_+ e^{-\gamma_p t_s})}$ where $\lambda_p = ab(1 - e^{-\gamma_p t_s})$, i.e.

$$F_+(q) = \sum_{n'_+} \int_0^\infty dt_s g(t_s) \left(\sum_{\tilde{n}_+} q^{\tilde{n}_+/2} p(\tilde{n}_+, t_s|n'_+) \right) P_+^{ss}(n'_+)$$
$$= \int_0^\infty dt_s g(t_s) \sum_{n'_+} P_+^{ss}(n'_+) q^{\frac{1}{2}(\lambda_p + n'_+ e^{-\gamma_p t_s})}$$
$$= \int_0^\infty dt_s g(t_s) q^{\frac{1}{2}\lambda_p} \sum_{n'_+} P_+^{ss}(n'_+) \left(q^{\frac{1}{2} e^{-\gamma_p t_s}} \right)^{n'_+}$$
$$= \int_0^\infty dt_s g(t_s) q^{\frac{1}{2}\lambda_p} F_+(q^{\frac{1}{2} e^{-\gamma_p t_s}}) \tag{76}$$

The moment $\langle n_+ \rangle = q\frac{\partial}{\partial q}F_+(q)\Big|_{q=1}$ and $\langle n_+^2 \rangle = q\frac{\partial}{\partial q}q\frac{\partial}{\partial q}F_+(q)\Big|_{q=1}$, and hence taking derivatives of on two sides of Eq. 74 and 76 respectively, and setting $q = 1$, we may obtain the moments in the two cases above. The explicit forms of CV^2 thus obtained are shown in Eq. 32 in the main text (corresponding to Eq. 74) and in Eq. 34 in the main text (corresponding to Eq. 76).

[1] K. Rijal, N. I. C. Müller, E. Friauf, A. Singh, A. Prasad, and D. Das, Phys. Rev. Lett. **132**, 228401 (2024).
[2] V. Shahrezaei and P. S. Swain, Proc. Natl. Acad. Sci. USA **105**, 17256 (2008).
[3] D. T. Gillespie, J. Phys. Chem. **81**, 2340 (1977).
